# Humanin-G Ameliorates Hemorrhage-Induced Acute Lung Injury in Mice Through AMPKα1-Dependent and -Independent Mechanisms

**DOI:** 10.3390/biomedicines12112615

**Published:** 2024-11-15

**Authors:** Allison M. Amman, Vivian Wolfe, Giovanna Piraino, Assem Ziady, Basilia Zingarelli

**Affiliations:** 1 Department of Surgery, University of Cincinnati College of Medicine, 231 Albert Sabin Way, Cincinnati, OH 45267, USA; ammannao@ucmail.uc.edu; 2 Department of Pediatrics, University of Cincinnati College of Medicine, Division of Critical Care Medicine, Cincinnati Children’s Hospital Medical Center, 3333 Burnet Avenue, Cincinnati, OH 45229, USA; vivian.xue@cchmc.org (V.W.); giovanna.piraino@cchmc.org (G.P.); 3 Department of Pediatrics, University of Cincinnati College of Medicine, Division of Bone Marrow Transplantation & Immune Deficiency, Cincinnati Children’s Hospital Medical Center, 3333 Burnet Avenue, Cincinnati, OH 45229, USA; assem.ziady@cchmc.org

**Keywords:** age, AMP-activated protein kinase (AMPK), hemorrhagic shock, sex, humanin-G, neutrophils, signal transducer and activator of transcription-3 (STAT3)

## Abstract

**Background/Objectives**: The severity of acute lung injury is significantly impacted by age and sex in patients with hemorrhagic shock. AMP-activated protein kinase (AMPK) is a crucial regulator of energy metabolism but its activity declines with aging. Humanin is a mitochondrial peptide that exerts cytoprotective effects in response to oxidative stressors and is associated with longevity. Using a mouse model of hemorrhagic shock that mimics the clinical condition of adult patients, we investigated whether treatment with a humanin analog, humanin-G, mitigates lung injury and whether its mechanisms of action are dependent on the catalytic AMPKα1 subunit activation. **Methods**: Male and female AMPKα1 wild-type (WT) and knock-out (KO) mice (8–13 months old) were subjected to hemorrhagic shock by blood withdrawal, followed by resuscitation with shed blood and lactated Ringer’s solution. The mice were treated with PEGylated humanin-G or vehicle and euthanized 3 h post-resuscitation. **Results**: Sex- and genotype-related differences were observed after hemorrhagic shock as lung neutrophil infiltration was more pronounced in the male AMPKα1 WT mice than the female WT mice; also, the male AMPKα1 KO mice experienced a significant decline in mean arterial blood pressure when compared to the male WT mice after resuscitation. The scores of histological lung injury were similarly elevated in all the male and female AMPKα1 WT and KO mice when compared to the control mice. At molecular analysis, acute lung injury was associated with the downregulation of AMPKα1/α2 catalytic subunits in the WT mice, whereas an increased activation of the signal transducer and activator of transcription-3 (STAT3) was observed in all the vehicle-treated groups. The in vivo administration of humanin-G ameliorated histological lung damage in all the groups of animals and ameliorated mean arterial blood pressure in the male AMPKα1 KO mice. The in vivo administration of humanin-G lowered lung neutrophil infiltration in the male and female AMPKα1 WT mice only but not in the KO mice. The beneficial results of humanin-G correlated with the lung cytosolic and nuclear activation of AMPKα in the male and female AMPKα1 WT groups, whereas STAT3 activation was not modified. **Conclusions**: In adult age, hemorrhage-induced acute lung injury manifests with sex-dependent characteristics. Humanin-G has therapeutic potential and the AMPKα1subunit is an important requisite for its inhibitory effects on lung leucosequestration, but not for the amelioration of lung alveolar structure or the hemodynamic effects of the peptide.

## 1. Introduction

Hemorrhagic shock is associated with a high incidence of acute lung injury (30% to 50%) and is a predictor of poor outcomes in trauma victims [1,2,3,4]. The risk of acute lung injury and the associated high mortality increase with the age of the patient [2,3]. Clinical studies also suggest that sex-dimorphic immunomodulatory and inflammatory responses impact the outcome of trauma adult victims [5,6,7,8]. For example, women are less susceptible to developing trauma-related complications, including lung injury, and have better survival rates than men with equivalent injuries [8,9]. The treatment of acute lung injury focuses on supportive ventilation, but effective pharmacotherapies are limited.

An important pathogenetic mechanism of organ injury in critical illness has been related to the impairment of mitochondrial function and the subsequent cellular failure to utilize oxygen despite the implementation of resuscitation protocols [10,11]. The AMP-activated protein kinase (AMPK) is a conserved regulator of cellular energy homeostasis and is activated upon a decrease in ATP and an increase in AMP. The catalytic α-subunit is expressed as α1 and α2 isoforms which display tissue-specific distribution. The catalytic α1 isoform is the most abundantly expressed in the lung [12]. Aging-associated loss in AMPK activity has been reported to contribute to mitochondrial dysfunction and to impact innate immune responses during stress [13,14]. In previous experimental studies, we have demonstrated that the age-dependent impairment of AMPK activation plays a pathogenic role in multiple organ injury in middle-aged male mice when compared with young animals in models of sepsis and hemorrhagic shock [15,16,17,18]. In our previous studies in murine models of severe hemorrhagic shock, the pharmacological activation of the AMPK pathway provided beneficial effects by moderating the inflammatory responses and by promoting metabolic recovery in injured organs [16,17,18].

Humanin is a mitochondrial peptide encoded by the MT-RNR2 gene located within the mitochondrial genome and originally isolated from a cDNA library of the surviving neurons of familial Alzheimer’s disease [19,20]. This peptide can be released extracellularly and is present in circulation and tissues in humans and experimental animals [21,22]. Experimental studies describe the potent cytoprotective effects of humanin and its synthetic derivatives. Humanin has been shown to have cytoprotective effects in endothelial cells against oxidative stress [23,24] and in models of neurodegenerative disorders and cerebral ischemia and reperfusion injury [25,26,27,28,29]. Some of humanin’s mechanisms of action have been attributed to the activation of the signal transducer and activator of transcription-3 (STAT3) [25,29,30]. Recently, we have also demonstrated that the potent synthetic derivatives, humanin-G and colivelin, provide beneficial effects in young mouse models of hemorrhagic shock and sepsis [22,31].

Because of the age- and sex-related incidence and mortality in trauma victims, in the present study, we investigated the therapeutic efficiency of a PEGylated form of humanin-G in an adult model of hemorrhagic shock using middle-aged mice (8–13 months), e.g., retired breeder mice at a stage where reproductive performance has ceased but senescence has not yet been fully reached [32]. To extend our understanding of the mechanisms that contribute to hemorrhage-induced acute lung injury, we also sought to determine the role of AMPKα1 and STAT-3 activation.

## 2. Materials and Methods

### 2.1. Model of Hemorrhagic Shock

The experiments conformed to the Guide for the Care and Use of Laboratory Animals published by the US National Institutes of Health (Eighth edition, 2011) and had the approval of the Institutional Animal Care and Use Committee of the Cincinnati Children’s Hospital Medical Center (protocol ID# IACUC2024-0009). The experiments were carried out in compliance with the ARRIVE guidelines. Male and female AMPKα1 wild-type (WT) and AMPKα1 knockout (KO) mice were generated on a C57/BL6 background strain [33]. The mice were housed in a temperature-controlled room at 21 ± 2 °C with a 14 h light/dark cycle and were allowed free access to water and a maintenance diet. At the age range of 8–13 months, old mice were subjected to hemorrhagic shock. The mice were anesthetized with Ketamine (90 mg/kg) and Xylazine (10 mg/kg) intraperitoneally and were placed on a circulating warming water blanket to maintain body temperature at physiological conditions throughout the experiment. Either the left or right femoral artery was cannulated (PE-10 tube) and connected to a blood pressure transducer (PowerLab, ADInstruments, Colorado Springs, CO, USA) for the measurement of mean arterial blood pressure (MABP) and heart rate (HR). Hemorrhagic shock was induced by blood removal until MABP reached 30 ± 5 mmHg [18,22]. The mice were kept in this MABP range for 90 min by additional blood removal or the transfusion of a small volume of shed blood. At the end of the shock period, the mice received the following resuscitation fluids over a 10 min period: their shed blood and lactated Ringer’s solution (two times the total volume of removed blood). The mice were then allocated to two treatment groups in a random fashion: the vehicle group received distilled water (500 µL); the treatment group received PEGylated humanin-G (100 µg/kg). Vehicle or PEGylated humanin-G was administered at the initiation of resuscitation via the femoral artery. The dose of the PEGylated humanin-G was selected from our previous studies demonstrating therapeutic effects in young mice subjected to hemorrhagic shock [22]. MABP and HR were further recorded for 3 h after resuscitation. The control mice were exposed to anesthesia only; these mice were not subjected to surgery or hemorrhage protocol. The mice were euthanized at 3 h after resuscitation for lung collection.

### 2.2. Preparation of PEGylated Humanin-G

The covalent conjugation of polyethylene glycol (PEG) (1:1 molar ratio) was used to generate a long-acting humanin-G. In brief, humanin-G was suspended in phosphate-buffered saline, pH 7.4, with 5 mM EDTA. This solution was combined with an equal volume of DMSO with PEG-maleimide and incubated overnight at room temperature. The PEGylated humanin-G (PEG10 kDa) was then filtered by size-exclusion chromatography with a 50 mM ammonium acetate buffer [34].

### 2.3. Histopathologic Analysis

Formalin-fixed and paraffin-embedded lung tissue blocks were sectioned at 5 μm thickness and were stained with hematoxylin and eosin. Lung injury was examined by light microscopy by four independent observers blinded to the treatment groups. Injury scores were calculated by a semiquantitative analysis based on the following histologic features: alveolar capillary congestion, the infiltration of red blood cells and inflammatory cells into the airspace, alveolar wall thickness, and hyaline membrane formation [31]. A score of 0 represented normal findings and scores of 1, 2, 3, and 4 represented minimal (<25% lung involvement), mild (25–50% lung involvement), significant (50–75% lung involvement), and severe (>75% lung involvement) injury, respectively. The four variables were summed to represent the lung injury score (total score, 0–16).

### 2.4. Lung Myeloperoxidase Assay

Myeloperoxidase (MPO) activity was determined as an index of neutrophil accumulation in the lung [31]. Lung tissues were homogenized in a solution containing 0.5% hexa-decyl-trimethyl-ammonium bromide dissolved in 10 mM 3-(morpholin-4-yl)propane-1-sulfonic acid (pH 7) and centrifuged for 30 min at 4000× *g* at 4 °C. An aliquot of the supernatant (5 µL) was allowed to react for 5 min with a solution (total volume 1 mL) by adding 80 mM sodium phosphate buffer (395 µL, pH 5.5) and 20 mM tetra-methyl-benzidine (50 µL in 100% dimethyl sulfoxide). At the end of the reaction period, 0.1 mM H_2_O_2_ solution was added for 3 min. The reaction was then halted by the addition of 2 M acetic acid (500 µL) at 4 °C. The rate of change in absorbance was measured by spectrophotometry at 650 nm. Myeloperoxidase activity was defined as the quantity of enzyme degrading 1 μmol of hydrogen peroxide/minute at 37 °C and expressed in units per 100 mg tissue.

### 2.5. Lung Levels of ATP

The content of ATP was evaluated in the lung after homogenization. A colorimetric ATP assay kit was used for measurement (BioVision, Milpitas, CA, USA) according to the recommended protocol.

### 2.6. Cytosol and Nuclear Extracts

Lung tissues were homogenized in a buffer containing 0.32 M sucrose, 10 mM TrisHCl (pH 7.4), 1 mM EGTA, 2 mM EDTA, 5 mM NaN3, 10 mM β-mercaptoethanol, 20 mM leupeptin, 0.15 mM pepstatin A, 0.2 mM phenylmethanesulfonyl fluoride, 50 mM NaF, 1 mM sodium orthovanadate, and 0.4 nM microcystin. The samples were centrifuged at 1000× *g* for 10 min at 48 °C and the supernatants were collected as cytosol extracts. The pellets were then solubilized in Triton buffer (1% Triton X-100, 250 mM NaCl, 50 mM Tris HCl at pH 7.5, 3 mM EGTA, 3 mM EDTA, 0.1 mM phenylmethanesulfonyl fluoride, 0.1 mM sodium orthovanadate, 10% glycerol, 2 mM p-nitrophenyl phosphate, 0.5% NP-40, and 46 mM aprotinin). The lysates were centrifuged at 15,000× *g* for 30 min at 48 °C and the supernatant was collected as nuclear extracts. The total content of proteins was determined by the Bradford assay.

### 2.7. Western Blot Analysis

The cytosol and nuclear content of both subunits AMPKα1/α2 and their phosphorylated active forms pAMPK α1/α2, and STAT3 and its two phosphorylated active forms pSTAT3(Tyr705) and pSTAT3(Ser727) were determined by immunoblot analyses. Glyceraldehyde-3-phosphate dehydrogenase (GADPH) and β-actin were measured as loading control proteins. Proteins (20 µg) were separated using electrophoresis on a 10% Bis-Tris gel and then transferred onto nitrocellulose membranes. Western blotting was performed using the IBind Flex Western System (Thermo Fischer Scientific, Waltham, MA, USA) that employs sequential lateral flow to achieve blocking and antibody binding. The Odyssey LI-COR scanner and Image Studio software version 6.0 (LI-COR Biotechnology, Lincoln, NE, USA) were used for the detection and quantitative analysis of the immunoblotting.

### 2.8. Materials and Reagents

Humanin-G ([Gly14]-Humanin) was purchased from AnaSpec (Fremont, CA, USA). The primary antibodies for AMPKα1/α2 and pAMPKα1/α2, STAT3, pSTAT3(Tyr705), and pSTAT3(Ser727), were purchased from Cell Signaling Technology (Danvers, MA, USA). The primary antibodies for GADPH and β-actin were purchased from Santa Cruz Biotechnology (Dallas, TX, USA). The Odyssey blocking buffer, LI-COR goat anti-rabbit IRDye-800CW, goat anti-mouse IRDye-680RD antibodies, and the 4X Protein Sample Loading Buffer were purchased from LI-COR Biotechnology (Lincoln, NE, USA). The IBind Western System solution kit was purchased from Thermo Fischer Scientific (Waltham, MA, USA). The NuPAGE LDS Sample Buffer and Western blot gels were purchased from Life Technologies (Grand Island, NY, USA). All the other chemicals were purchased from Sigma-Aldrich (St. Louis, MO, USA).

### 2.9. Statistical Analysis

All the statistical analyses were performed with SigmaPlot for Windows 14.5 (Systat Software, San Jose, CA, USA). Data are reported as the means ± SEM of *n* = 4–7 animals for each age and sex group. The one-way analysis of variance (ANOVA) with Student–Newman–Keuls correction was adopted for multiple group analysis at a single time point. A two-way ANOVA with Student–Newman–Keuls correction was adopted for multiple group analysis at different time points. A Mann–Whitney Rank Sum test or an ANOVA on ranks test was performed when the data failed normal distribution. *p*-values less than 0.05 were considered significant.

## 3. Results

### 3.1. In Vivo Administration of PEGylated Humanin-G Ameliorates MABP in Male AMPKα1 KO After Hemorrhagic Shock

**Figure 1:**
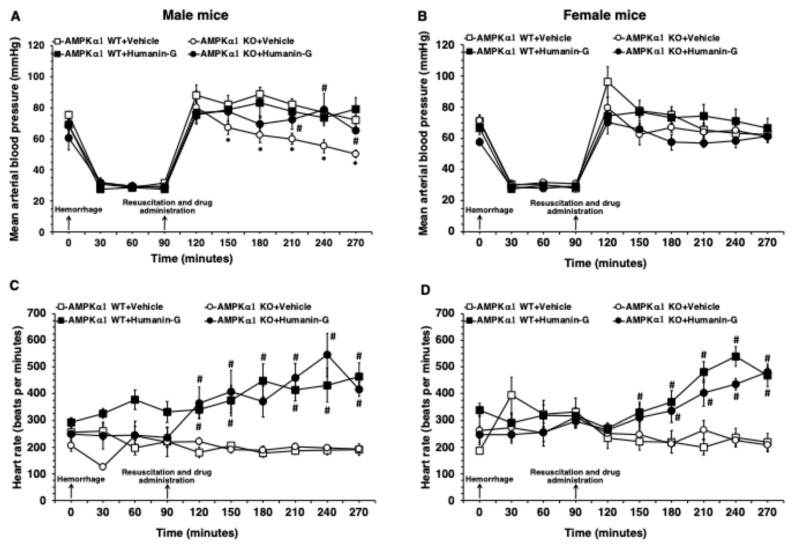
Effect of the in vivo administration of PEGylated humanin-G on (**A**,**B**) mean arterial blood pressure and (**C**,**D**) heart rate in the male and female AMPKα1 wild-type (WT) and knockout (KO) mice subjected to hemorrhage and resuscitation. Data represents the mean ± SEM of 4–7 mice in each group. Vehicle or PEGylated humanin-G (100 µg/kg) was given intra-arterially at the initiation of resuscitation. Arrows indicate the time of the induction of hemorrhage and initiation of resuscitation and administration of PEGylated humanin-G or vehicle. * *p* < 0.05 versus vehicle-treated AMPKα1-WT group; # *p* < 0.05 versus vehicle-treated group of the same genotype.

Since the degree of hypoperfusion influences the severity of the inflammatory response, we chose an experimental protocol of pressure-controlled hemorrhage to a target MABP of 30 mmHg in both sex groups [17]. MABP fully recovered in the AMPKα1 WT and KO female mice and WT male mice after the vehicle treatment and fluid resuscitation (Figure 1A,B and Figure S1). However, the vehicle-treated male AMPKα1 KO animals experienced a progressive and significative decline of MABP when compared to the male WT mice (Figure 1A). There was no statistical difference in HR in the vehicle-treated male or female AMPKα1 WT and KO mice throughout the experimental period. Treatment with PEGylated humanin-G at the time of resuscitation significantly improved MABP in the male AMPKα1 KO mice when compared with the vehicle treatment (Figure 1A). The administration of PEGylated humanin-G did not affect MABP in the AMPKα1 male WT mice and in the female WT and KO groups. HR was significantly higher after humanin-G treatment in both the male and female AMPKα1 WT and KO mice when compared to the vehicle treatment (Figure 1C,D and Figure S1).

### 3.2. In Vivo Administration of PEGylated Humanin-G Improves Lung Architecture, but Not Neutrophil Infiltration, in an AMPKα1-Independent Manner

**Figure 2:**
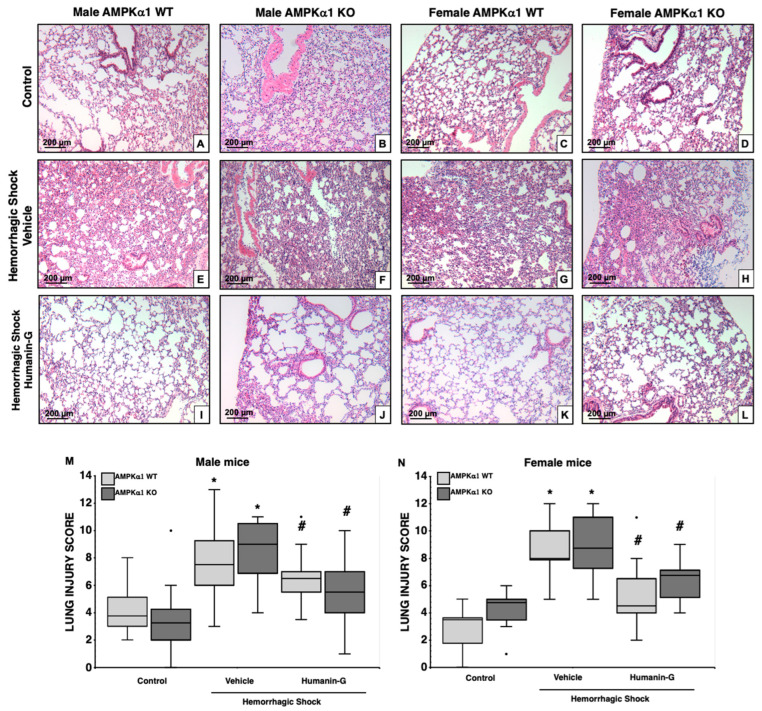
Effect of the in vivo administration of PEGylated humanin-G on lung injury in the male and female AMPKα1 wild-type (WT) and knockout (KO) mice subjected to hemorrhage and resuscitation. The representative histology photomicrographs of (**A**–**D**) sections of the control male and female mice showing normal lung architecture; (**E**–**H**) sections of the vehicle-treated male and female mice showing reduction in alveolar space and the marked infiltration of inflammatory cells; (**I**–**L**) sections of the PEGylated humanin-G-treated male and female mice showing the amelioration of lung architecture in all the groups but (**J**–**L**) persistence of inflammatory cells (arrows) in the male and female KO mice. Magnification ×100. A similar pattern was seen in n = 4 different tissue sections in each experimental group. (**M**,**N**) The histopathologic scores of lung sections (n = 4 mice for each group). Lung injury was scored from 0 (no damage) to 16 (maximum damage). Box plots represent 25th percentile, median, and 75th percentile; error bars define 10th and 90th percentiles. * *p* < 0.05 versus sex- and genotype-matched control; # *p* < 0.05 versus vehicle-treated group of the same genotype.

At 3 h after resuscitation, histological analysis revealed that all the vehicle-treated groups had a similar degree of total lung injury score as more than 75% of the lung sections were involved (Figure 2). The lung histology of the vehicle-treated mice subjected to hemorrhagic shock was characterized by large hemorrhagic areas, marked neutrophil margination along vessel walls, and reduction in alveolar air space (Figure 2E–H) when compared to the lungs of the sex- and genotype-matched control mice (Figure 2A–D). After treatment with PEGylated humanin-G, all the male and female AMPKα1 WT and KO mice had a significantly decreased lung injury score compared to the vehicle-treated groups. Interestingly, although treatment with PEGylated humanin-G appeared to improve alveolar space (Figure 2I–L) and to reduce total damage score in all the groups (Figure 2M,N), there was still a persistent neutrophil infiltration in both the male and female KO mice at histological analysis (Figure 2I–L).

**Figure 3:**
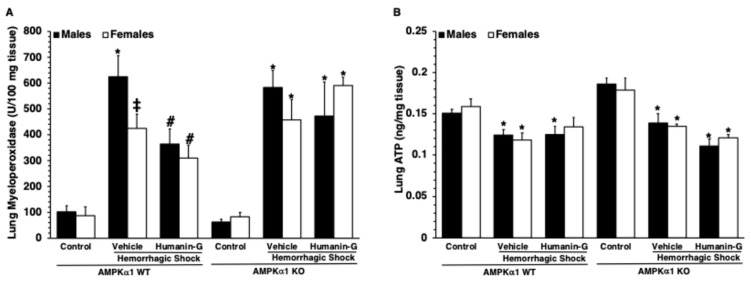
Effect of the in vivo administration of PEGylated humanin-G on (**A**) lung neutrophil infiltration and (**B**) lung ATP levels in the male and female AMPKα1 wild-type (WT) and knockout (KO) mice subjected to hemorrhage and resuscitation. Data are expressed as the means ± SEM of 4–7 mice in each group. Vehicle (distilled water) or PEGylated humanin-G (100 µg/kg) was given intra-arterially at the initiation of resuscitation. * *p* < 0.05 versus control group of the same genotype; # *p* < 0.05 versus vehicle-treated group of the same genotype; ‡ *p* < 0.05 versus the AMPKα1-WT male group.

To confirm the extent of neutrophil infiltration, we measured the content of MPO, a heme-containing peroxidase expressed mainly in neutrophils. Lung MPO content increased in all the vehicle-treated groups after hemorrhagic shock when compared to the sex- and genotype-matched controls (Figure 3A). The male AMPKα1 WT mice also had higher MPO levels than the WT female mice. The administration of the PEGylated synthetic peptide notably decreased MPO levels in the male and female AMPKα1 WT mice compared to the vehicle treatment but did not affect neutrophil infiltration in the KO mice (Figure 3A), thus confirming the histological findings (Figure 2I–L).

### 3.3. In Vivo Administration of PEGylated Humanin-G Did Not Modify Content of Lung ATP

To determine whether treatment with PEGylated humanin-G might affect energy homeostasis in the lung, we measured ATP levels. There was no statistical difference in ATP levels among the control male or female AMPKα1 WT and KO mice at baseline conditions. At 3 h after resuscitation, the ATP levels significantly decreased in the lung tissue of the vehicle-treated mice of all the groups in comparison to the baseline content of the sex- and genotype-matched control mice (Figure 3B). The administration of PEGylated humanin-G did not improve the ATP levels in any of the experimental groups.

### 3.4. In Vivo Administration of PEGylated Humanin-G Restores Activation of AMPKα1 in the Lung

**Figure 4:**
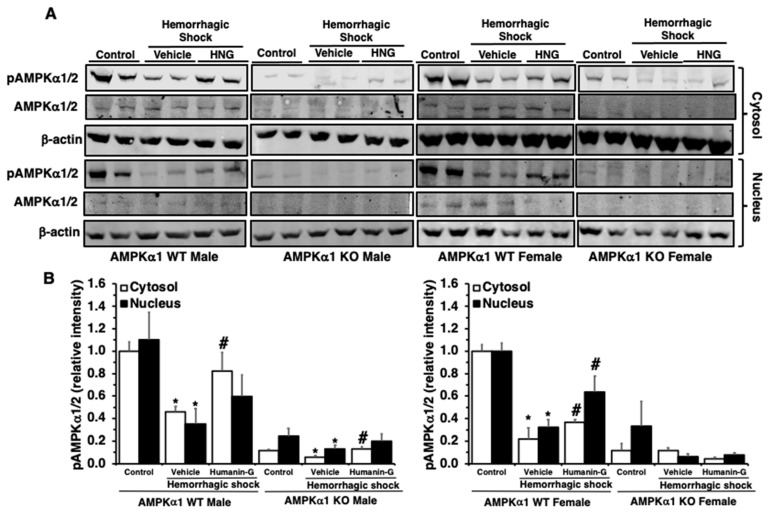
(**A**) Illustrative Western blots of p-AMPKα1/2, total AMPKα1/2, and β-actin (used as control protein) in the cytosol and nuclear extracts of the lungs of the male and female AMPKα1 wild-type (WT) and knockout (KO) mice subjected to hemorrhage and resuscitation. (**B**) The densitometric analysis of p-AMPKα1/2 expression. Data are the means of n = 4–7 mice for each group. Vehicle or PEGylated humanin-G (100 µg/kg) was given intra-arterially at the initiation of resuscitation. * *p* < 0.05 versus control group of the same genotype; # *p* < 0.05 versus vehicle-treated group of the same genotype.

Since PEGylated humanin-G reduced lung neutrophil infiltration only in the presence of a functional AMPKα1 gene in the WT mice, next we sought to investigate the effect of PEGylated humanin-G on the expression of the catalytic subunits α1 and α2 of AMPK (Figure 4). At 3 h after resuscitation, immunoblotting with antibodies for both AMPKα1/2 revealed a decrease in the expression of the active pAMPKα1/2, but not the total AMPKα1/2, in the cytosol and nucleus in the vehicle-treated WT mice of both sexes when compared to basal condition, further confirming our previous studies showing that in hemorrhagic shock there is an age-dependent impairment of AMPK activation [16,17,18]. The administration of the PEGylated peptide augmented the expression of pAMPKα1/2 in both the cytosol and nucleus of the WT mice of both sexes. In the AMPKα1 KO mice, immunoblotting revealed no band or a very faint one representing most probably the AMPKα2 subunit. We noted only a cytosol downregulation of the pAMPKα2 band in the vehicle-treated male KO mice; the PEGylated humanin-G treatment partially restored the levels of pAMPKα2 in the male KO mice. Thus, these findings confirmed that AMPKα1 is the most abundant subunit in the lung and that the protective effects of PEGylated humanin-G are mediated by AMPKα1 activation.

### 3.5. In Vivo Administration of PEGylated Humanin-G Does Not Modify Activation of STAT3 in the Lung

**Figure 5:**
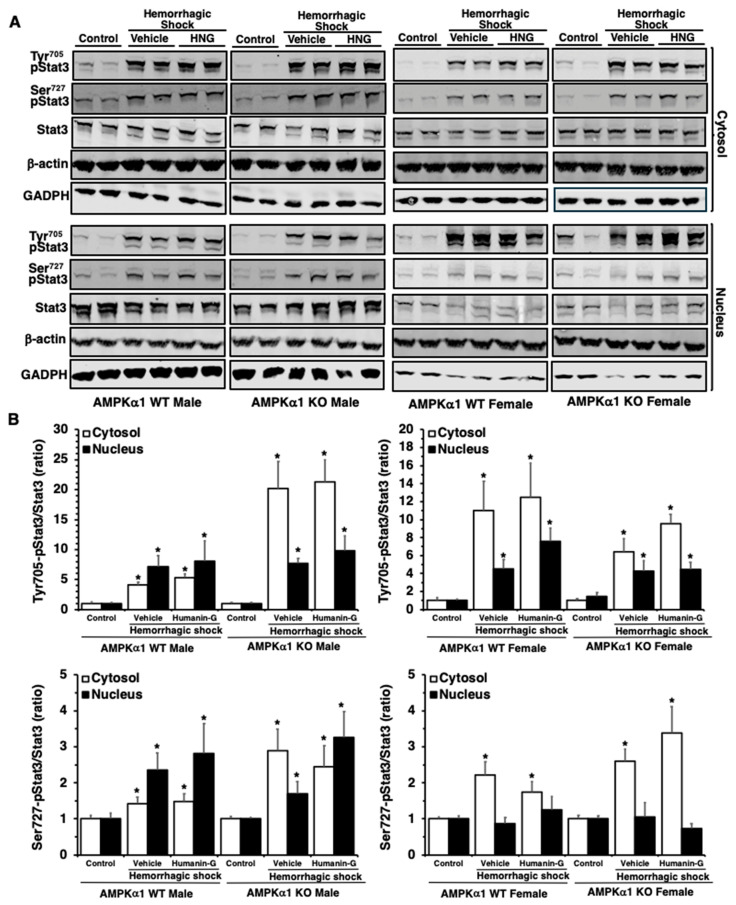
(**A**) Illustrative Western blots of pSTAT3(Tyr705), pSTAT3(Ser727), total STAT3, β-actin, and GADPH (used as control proteins) in the cytosol and nuclear extracts of the lungs of the male and female AMPKα1 wild-type (WT) and knockout (KO) mice subjected to hemorrhage and resuscitation. (**B**) The densitometric analysis of the relative intensity ratio of p-STAT3(Ser727)/STAT3, and the ratio of p-STAT3(Tyr705)/STAT3. Data are the means of n = 4–7 mice for each group. Vehicle or PEGylated humanin-G (100 µg/kg) was given intra-arterially at the initiation of resuscitation. * *p* < 0.05 versus control group of the same genotype.

Since the molecular mechanisms of mitochondrial peptides have also been ascribed to the activation of STAT3, an important transcription factor in inflammation, immunity, and metabolism [29], we next investigated whether the molecular mechanisms of PEGylated humanin-G were correlated with the changes in the activation of STAT3 in the lung. The control AMPKα1 WT and KO mice displayed slight levels of pSTAT3(Ser727) and pSTAT3(Tyr705) in the cytosol and nucleus (Figure 5). At 3 h after resuscitation, the expression of both pSTAT3(Ser727) and pSTAT3(Tyr705) was augmented in the cytosol and nucleus in the male and female mice of both genotypes in comparison to the basal expression of the sex-matched control mice, suggesting a global cellular activation of this transcription factor (Figure 5). There were no changes in the total content of STAT3 among all the experimental groups in either the WT or KO mice. The administration of PEGylated humanin-G did not change the cytosolic or nuclear expression of pSTAT3(Tyr705) or pSTAT3(Ser727) in any groups of animals (Figure 5).

## 4. Discussion

This is the first study, to our knowledge, to demonstrate the therapeutic potential of humanin-G, a potent synthetic humanin analog, to reduce hemorrhage-induced lung injury in middle-aged mice when administered in addition to the standard blood and fluid resuscitation. In our study, the therapeutic effects of the synthetic analog of humanin were observed in both male and female AMPKα1 WT and KO mice, although with different histological characteristics, thus supporting the involvement, at least in part, of AMPKα1-independent mechanisms.

Sex dimorphism in multiple organ failure incidence and related death has been observed after trauma in adult populations. Specifically, the male gender appears to be an independent prognostic variable for trauma-associated higher risks of infections, the development of complications, and negative outcomes [5,7,8,9]. Among the adult population, aging also strongly correlates with high morbidity and mortality and appears to be a risk factor for poor outcomes independently of injury severity after severe trauma with hemorrhagic shock [2,3,4]. The presence of acute lung injury is a predictor of poor outcome in trauma patients as it is associated with multiple organ dysfunction syndrome and high mortality rates [1]. Therefore, understanding pathophysiological mechanisms and developing novel interventions, which could prevent acute lung injury, have the potential to reduce the substantial morbidity, mortality, and resource utilization associated with this syndrome.

Previous studies from our laboratory demonstrated that AMPK is an important pathway to regulate energy homeostasis under the pathological events of multiple organ failure [15,16,17,18]. We demonstrated that the kinase becomes dysregulated with aging and this impairment plays a pathogenetic role in myocardial and lung injury in middle-aged male mice after hemorrhagic shock [16,17,18]. Interestingly, our previous data have also shown that young KO mice with a genetic deficiency of the subunit AMPKα1 acquire an aging phenotype with the worsening of organ failure and mortality in sepsis and hemorrhagic shock and they cannot be rescued by AMPK activators, confirming the important role of this kinase [15,18]. To further support the role of AMPK in an aging population and the impact of sex as a biological variable, we sought to extend these initial results in middle-aged male and female AMPKα1 WT and KO mice. Interestingly, in contrast to the remarkable differences in the severity of organ injury seen in the young models of hemorrhagic shock [18,22,35] between the KO and WT mice of both sexes, differences in hemodynamics and lung neutrophil infiltration manifested in a sex-dependent manner in the middle-aged KO and WT mice. We observed that the genetic absence of AMPKα1 worsened hemodynamic decompensation in the middle-aged male AMPKα1 KO mice only but not in the female KO mice when compared to the WT mice. Nevertheless, despite this worse hemodynamic decompensation, the genetic absence of AMPKα1 in the KO male mice did not correlate with differences in lung injury when compared to the WT mice. Given that AMPKα1 expression declines with age, it is possible that the additional effects of age-related AMPK dysregulation in the middle-aged WT mice have resulted in less robust phenotypic effects in organ injury relative to the KO-type mice. Nevertheless, consistent with previous reports demonstrating sex dimorphism in hemorrhagic shock [3,4,5,6,7], in our study, we demonstrated that the male AMPKα1 WT mice had significantly higher lung neutrophil infiltration than the female WT mice. However, the sex-specific differences observed in the WT mice were abrogated in the AMPKα1 KO mice, as the KO females experienced similar lung neutrophil infiltration as the KO male mice after hemorrhagic shock, suggesting that AMPKα1 could be one of the biological factors mediating the resilience of females to lung injury. An interesting finding of our study is that sex-related differences were found in 8–13-month-old mice. At this age, both male and female mice are considered retired breeders, which have passed their peak reproductive age, most probably with declining levels of sex hormones [32]. Therefore, our data would argue against the involvement of estrogens in the less severe inflammation in the lungs of the female AMPKα1 WT mice when compared to the male WT mice. Furthermore, this mouse model is relevant to epidemiological studies with patient populations > 50 years old. It is important to note that in clinical studies, the independent protective effect of female gender on multiple organ failure remains significant in both premenopausal and postmenopausal women when compared with similarly aged men [7]. This is contrary to previous experimental studies and the known physiologic sex hormone changes that occur after menopause in women [5]. Thus, taken together, our findings support the notion that the effects of AMPKα1 may contribute to regulating inflammation in a sex-dependent manner. Further studies are required to clarify the role of AMPKα1 with respect to sex-related protective mechanisms.

In this study, we tested the efficacy of humanin-G, a synthetic analog of the mitochondrial peptide humanin. Humanin is a 24-amino acid peptide that was initially isolated from a neuronal cDNA library of patients with Alzheimer’s disease [20]. Various in vitro experimental studies describe the potent cytoprotective effects of humanin and its analogs in oxidant-induced endothelial cell damage [23] and in age-related neuronal cell death [26,27,28]. Furthermore, in vivo treatment with humanin has been shown to ameliorate tissue damage in experimental cerebral [28] and myocardial ischemia and reperfusion injury [36]. Consistent with these therapeutic effects of the peptide, we have previously demonstrated that the in vivo administration of the synthetic analog humanin-G, which has stronger potency than the precursor humanin [37], ameliorated hemodynamic parameters, organ damage, and survival of young female mice exposed to severe hemorrhage. In the current study, we further demonstrated that humanin-G treatment can afford beneficial effects in older ages. Furthermore, we propose that humanin-G may help to reduce damage in conditions of AMPKα1 deregulation as both the AMPKα1 WT and KO mice experienced lung protective effects.

Maladaptive decompensatory hypotension is a deleterious event of severe hemorrhage that leads to insufficient oxygen delivery to organs contributing to multiple organ failure [38]. While tachycardia is an important early sign of shock in trauma, older patients are less able to mount a tachycardia in response to decreased stroke volume because of decreased beta-adrenergic receptors in the heart and a depressed pacemaker function of the sinoatrial node [39,40]. In our study, we adopted a model of pressure-controlled hemorrhage to avoid differences in hypoperfusion [41]. We observed that all the groups were unable to mount a tachycardic response to the severe hemorrhage. However, the male and female vehicle-treated AMPKα1 WT mice were able to maintain their post-resuscitation blood pressure at normotension. In contrast, the vehicle-treated male, but not the female KO groups, experienced early and prolonged hypotension, further suggesting a role for AMPKα1 in female sex-dependent resilience in hemorrhagic shock. Treatment with humanin-G provided significant improvement in blood pressure in the male AMPKα1 KO mice. These data would imply that the re-establishment of preload and effective circulatory volume is mediated by the peptide in an AMPKα1-independent manner. Additionally, treatment with humanin-G significantly increased heart rate in all the male and female WT and KO groups when compared to the vehicle treatment, possibly reflecting an effect on the reflex control of heart rate. In support of these cardioprotective effects of humanin-G, a recent study has reported that chronic treatment with humanin-G prevented the progression of cardiac dysfunction and structural remodeling in a mouse model of heart failure [42].

A surprising finding of our study was a dissociation between improvement in the lung morphology and architecture and the effects on neutrophil infiltration in AMPKα1 KO mice after the treatment with humanin-G. We showed that the humanin G treatment resulted in significantly reduced hemorrhagic areas and alveolar disruption in the male and female AMPKα1 KO mice but, surprisingly, had no effect on neutrophil infiltration. Also, it appeared that humanin-G did not exert any modulatory role in the total content of ATP in the lung. Therefore, our data suggest that the major effect of humanin-G on lung neutrophil infiltration might be mediated by the activation of AMPKα1, whereas other AMPKα1-independent pathways may be involved in the protective effects of the mitochondrial peptide on the alveolar structure. To further understand the molecular mechanisms of humanin-G, we also investigated the activation of both AMPKα1 and α2 catalytic subunits. In our study, we observed that in the lung tissue, the AMPKα1 is the most expressed catalytic and its active phosphorylated form is downregulated after hemorrhagic shock. Remarkably, the humanin-G-treated mice had an increased activation of AMPK. Consistent with these data, we have previously demonstrated that treatment with colivelin, another humanin derivative, exerted pulmonary beneficial effects and reduced organ neutrophil infiltration in experimental septic shock by the activation of AMPKα1 in the aorta and lung [31]. Other studies have proposed that humanin and humanin analogs may exert beneficial effects on oxidative stress by the activation of AMPK [36,43].

Humanin-G has been shown to promote metabolic homeostasis [21,23,24,25]. In this current project, the in vivo administration of humanin-G was ineffective in restoring ATP levels in the lung after hemorrhagic shock. Nevertheless, we cannot rule out that humanin-G could exert beneficial effects on mitochondrial bioenergetics in other tissues. For example, we have previously demonstrated that the administration of humanin-G ameliorated mitochondrial structure and improved ATP content in cardiac tissue in young female mice subjected to hemorrhagic shock. This beneficial metabolic effect in the heart was associated with the amelioration of lung damage [22]. Therefore, additional investigation of mitochondrial oxidative phosphorylation function would be important to determine whether humanin-G may affect metabolic profile in energy-scarce conditions.

In further evaluating the molecular mechanisms of humanin-G, we also evaluated the role of STAT3, a transcription factor involved in the regulation of several immunological and inflammatory functions, and cell apoptosis [44]. Several in vitro studies have reported that humanin and its analogs may exert cytoprotective effects through STAT3 phosphorylation [29,30]. In our current study, we noted that the expression of pSTAT3(Tyr705) and pSTAT3(Ser727) was increased in the cytosol and nuclear compartments of the lung after hemorrhagic shock in all the mice that received vehicle. The in vivo administration of humanin-G did not modify the activation of STAT3 in the lungs. These data contrast with our previous findings in young mice subjected to hemorrhagic shock [22], where lung protective effects were associated with increased STAT3 activation. Although the physiologic significance of STAT3 in the lung is yet to be determined, the differential trends in these data suggest that STAT3 exerts different effects on the lung’s innate immune and inflammatory response in middle-aged mice compared to young mice during hemorrhagic shock. Taken together, these data also suggest that humanin-G could target different molecules in an age-dependent manner. For example, mitochondrial peptides have been described to bind with several cell surface receptors, such as formylpeptide-like-1 receptor and insulin-like growth factor binding protein-3, thus affecting other signaling pathways than AMPK or STAT3 [45]. Therefore, other investigations are required to define the exact AMPKα1-independent molecular mechanisms of humanin-G.

## 5. Conclusions

In conclusion, our data suggest that in adult age, hemorrhage-induced lung injury manifests with sex-dependent characteristics. The synthetic mitochondrial peptide humanin-G exerts hemodynamic and lung protective effects and may represent a therapeutic strategy in an aging population. The molecular mechanisms of action of humanin-G in the lung appear to be complex and to involve, at least in part, the activation of AMPKα1 but not STAT3 signaling. Whether the therapeutic effects of humanin-G are also secondary to the activation of multiple restorative metabolic pathways or interference with sex-related pathways needs to be further investigated.

## Data Availability

The data presented in this study are available upon request to the corresponding author.

## Supplementary Materials

The following supporting information can be downloaded at https://www.mdpi.com/article/10.3390/biomedicines12112615/s1, Figure S1: Effect of the in vivo administration of PEGylated humanin-G on (**A**) mean arterial blood pressure and (**B**) heart rate in male and female AMPKα1 wild-type (WT) and knockout (KO) mice subjected to hemorrhage and resuscitation.
