# Supplementary material for: Humanin-G Ameliorates Hemorrhage-Induced Acute Lung Injury in Mice Through AMPKα1-Dependent and -Independent Mechanisms
Source: Biomedicines. 2024 Nov 15;12(11):2615. doi: 10.3390/biomedicines12112615 (PMC11592305; doi:10.3390/biomedicines12112615)
Supplement: Supplementary file 1 [file biomedicines-12-02615-s001.zip › biomedicines-3241257-supplementary.pdf]

biomedicines

MDPI

Article

Humanin-G Ameliorates Hemorrhage-Induced Acute Lung Injury in Mice Through AMPKα1-Dependent and -Independent Mechanisms

Allison M. Amman [1], Vivian Wolfe [2], Giovanna Piraino [2], Assem Ziady [3] and Basilia Zingarelli [2,*]

[1] Department of Surgery, University of Cincinnati College of Medicine, 231 Albert Sabin Way, 45267 Cincinnati Ohio, USA; ammannao@ucmail.uc.edu
[2] Department of Pediatrics, University of Cincinnati College of Medicine, Division of Critical Care Medicine, Cincinnati Children's Hospital Medical Center, 3333 Burnet Avenue, 45229 Cincinnati OH, USA; vivian.xue@cchmc.org (V.W.); giovanna.piraino@cchmc.org (G.P.); basilia.zingarelli@cchmc.org (B.Z.)
[3] Department of Pediatrics, University of Cincinnati College of Medicine, Division of Bone Marrow Transplantation & Immune Deficiency, Cincinnati Children's Hospital Medical Center, 3333 Burnet Avenue, 45229 Cincinnati OH, USA; assem.ziady@cchmc.org
* Correspondence: basilia.zingarelli@cchmc.org

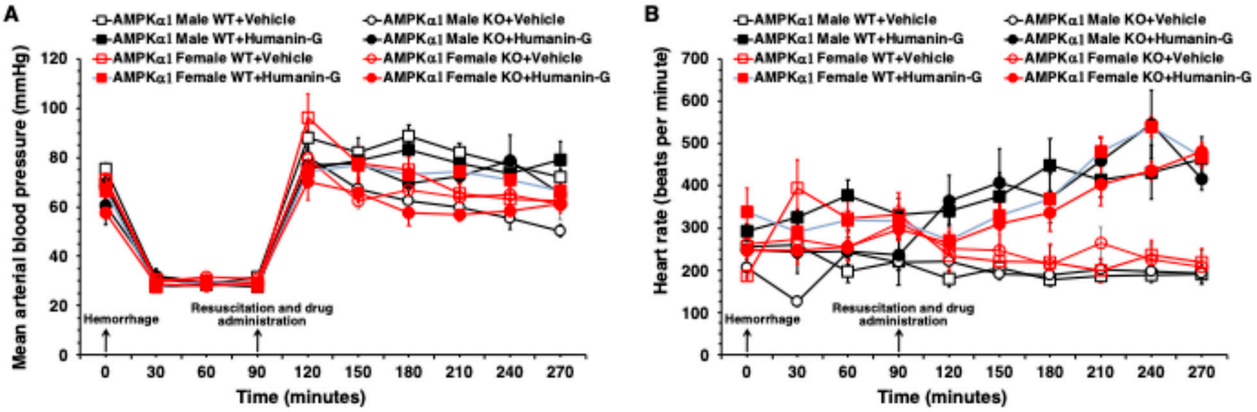

Figure S1. Effect of the in vivo administration of PEGylated humanin-G on (**A**) mean arterial blood pressure and (**B**) heart rate in male and female AMPKα1 wild-type (WT) and knockout (KO) mice subjected to hemorrhage and resuscitation. Data represents the mean ± SEM of 4-7 mice in each group. Vehicle (distilled water) or PEGylated humanin-G (100 μg/kg) was administered intra-arterially at the time of resuscitation. Arrows indicate time of induction of hemorrhage, and initiation of resuscitation and administration of PEGylated humanin-G or vehicle.
